# A survey of artificial intelligence in rheumatoid arthritis

**DOI:** 10.2478/rir-2023-0011

**Published:** 2023-07-22

**Authors:** Jiaqi Wang, Yu Tian, Tianshu Zhou, Danyang Tong, Jing Ma, Jingsong Li

**Affiliations:** Research Center for Healthcare Data Science, Zhejiang Laboratory, Hangzhou 311121, Zhejiang Province, China; Engineering Research Center of EMR and Intelligent Expert System, Ministry of Education, Key Laboratory for Biomedical Engineering of Ministry of Education, College of Biomedical Engineering and Instrument Science, Zhejiang University, Hangzhou 310027, Zhejiang Province, China

**Keywords:** rheumatoid arthritis, artificial intelligence, early diagnosis, early intervention, disease management

## Abstract

The article offers a survey of currently notable artificial intelligence methods (released between 2019-2023), with a particular emphasis on the latest advancements in detecting rheumatoid arthritis (RA) at an early stage, providing early treatment, and managing the disease. We discussed challenges in these areas followed by specific artificial intelligence (AI) techniques and summarized advances, relevant strengths, and obstacles. Overall, the application of AI in the fields of RA has the potential to enable healthcare professionals to detect RA at an earlier stage, thereby facilitating timely intervention and better disease management. However, more research is required to confirm the precision and dependability of AI in RA, and several problems such as technological and ethical concerns related to these approaches must be resolved before their widespread adoption.

## Introduction

Rheumatoid arthritis (RA) is a complex chronic autoimmune disease characterized by persistent inflammation with unclear etiology. It affects many joints, including the hands, feet, and wrists, and also causes pericarditis, pulmonary fibrosis, peripheral nephropathy, *etc*.^[1,2]^ The disease is associated with considerable morbidity, disability, and mortality, posing a significant burden on the mental level, physical level and social well-being of the patients.^[3]^ While there have been notable advancements in treatment, there is room for improvement in early diagnosis, timely treatment, and effective disease management. The slow progression and nonspecific initial symptoms of RA hinder accurate and prompt diagnosis. It is crucial to enhance the performance of diagnostic tools and the identify reliable biomarkers for early detection. Timely intervention is essential to delaying disease progression and minimizing irreversible joint damage. Challenges include drug selection difficulties, low treatment adherence, and patient variability in medication response. There is a need for personalized precision medicine systems and biomarkers to predict drug efficacy. Additionally, RA is a chronic condition that requires long-term management to control symptoms, prevent joint damage, and improve overall quality of life. However, poor treatment adherence, low follow-up rate, and inadequate chronic disease management contribute to a high disease recurrence rate. Therefore, extensive efforts in early diagnosis, timely intervention, prediction of disease progression, and disease management are important to improve the long-term outcomes of RA patients.

Artificial intelligence (AI) is defined as computers and other technology that mimic human intelligence-assisted mechanisms such as thinking, deep learning, reasoning, and sensory understanding.^[4]^ Machine learning, a subgroup of AI, is a powerful analytic approach, as stated by Dr. Stoel in his article, that is developed to automatically learn from the data and make decisions.^[5]^ In recent years, AI and its subclass machine learning (ML) have gained much attention as analytic methods and advanced techniques to improve efficacy and efficiency in healthcare. The medical AI/ML techniques are ranging from simple online appointment booking, health mentoring to complex drug development, disease diagnostics, digital consultation, and personalized treatment, *etc*.^[3]^ Moreover, the AI/ML systems have shown promise in various data types, such as electronic health records (EHRs), randomized controlled trials (RCTs), medical images and so on.^[4]^ For example, the medical AL/ML algorithms have been used to assist the selection of an appropriate antibiotic prescription based on the large number of patient’s demographic information and clinical history.^[6]^ Additionally, decision-making based on ML improves the accuracy and efficiency of decision-making by physicians.^[3]^ Medical AI techniques have been applied across various diseases including RA, including medical applications such as image analysis and diagnosis. These efforts illustrate that AI approaches have the potential to transform the treatment of rheumatic diseases. However, the use of AI in rheumatology is not as mature as in other medical areas. More studies are needed on the development and implementation of medical AI techniques to help diagnose and manage rheumatic diseases.^[7]^

We conducted an online search using PubMed in March 2023 using the following keywords “rheumatoid arthritis” and “artificial intelligence”. The study selection was limited to full-text studies published between 2019 to 2023 and the aim was related to the diagnosis, intervention, and management of RA. Our objective is to provide a survey of recent studies that have employed AI in RA, along with considering the potential role that AI might play in the future. Specifically, we will focus on key analyses, advanced methods, relevant strengths and limitations, and future directions in the following areas of RA: (1) diagnosis; (2) intervention; and (3) disease management.

## AI in the Diagnosis of RA

As the condition of RA progress, structural damage becomes a primary factor lead to functional disability.^[8]^ Late diagnosis is highly correlated with adverse long-term outcomes for RA patients. Therefore, the timely diagnosis of RA is crucial for the treatment of patients to better manage and improve the outcome of the patient.^[2]^ However, there are many barriers that existed which lead to delayed diagnosis of RA. One of the main difficulties is that signs and symptoms of RA in an early stage can be nonspecific, such as joint pain and stiffness, which can also be caused by other conditions.^[9]^ Furthermore, the progression of the disease can vary widely between individuals, making it difficult to identify and diagnose in its early stages.^[9]^ Another challenge is that there is no definitive test for RA. Diagnosis typically involves a combination of clinical evaluation, imaging tests, and laboratory tests, such as rheumatoid factor (RF) and anti-cyclic citrullinated peptide (anti-CCP) antibodies.^[2]^ However, these tests can have false results, which can lead to delayed or incorrect diagnoses. Therefore, there are significant challenges associated with early diagnosis of RA, emphasizing the critical importance of early detection for initiating timely treatment and preventing the progression to chronic RA.

Many machine learning works have been conducted in the diagnosis of RA to aid clinicians in making more accurate and efficient diagnoses (Table 1). First, AI algorithms, such as naive Bayes, convolutional neural network (CNN), logistic regression, and support vector machine (SVMs) or deep learning can analyze imaging data, such as X-rays, magnetic resonance imaging (MRI), and computer tomography (CT), to detect subtle changes in the joints that may indicate early RA.^[10,11]^ MRI can provide accurate data on early signs of inflammatory arthritis, particularly in the identification of bone marrow edema and tenosynovitis.^[12]^ X-rays is also most commonly used and easily accessible non-invasive method for monitoring disease progression, and can also detect bone structural changes in early stages.^[13]^ Bai *et al*.^[14]^ developed a method for detecting finger joint involvement in patients with RA using hand X-ray images, and they achieved an accuracy of 91.8% by training an artificial neural network (ANN) for this task. The automated analysis helps rheumatologists in the diagnosis process, replacing previous manual inspection of clinical images, which have greatly increased the efficacy and efficiency in the classification of RA.

**Table 1:** Summary of the studies of artificial intelligence (AI) in early diagnosis of rheumatoid arthritis (RA), including authors, data types, methods, and main findings of the same category

Main findings	Data types	Methods	Author (Month/Year)
Studies have demonstrated the effectiveness of CNN, Bayesian, or deep	Radiographs	CNN	Hirano *et al*.^[17]^ (Nov. 2019)
learning in detecting and classifying RA from radiographs. ML models have been developed to quantify radiographic joint damage, discriminate between RA, osteoarthritis and normal hand radiographs, and		CNN, DL	Üreten *et al*.^[18,19]^ (Apr. 2020, 2022)
detect joint ankylosis and subluxation. In addition, deep learning-based		Bayesian	Sun *et al*. ^[20]^ (Aug. 2022)
systems have been proposed for automatic assessment of bone destruction			
and computer-aided diagnosis of RA using hand radiographs. These		DL	Izumi *et al*.^[21]^ (Feb. 2023)
results demonstrate the potential of ML to improve the diagnosis and assessment of RA through automated image analysis.		DNN	Miyama *et al*.^[22]^ (Oct. 2022)
		DL	Wang *et al*.^[23]^ (Jun. 2022)
These studies focus on the use of ML, specifically deep learning or CNN, in analyzing ultrasound images for RA. They include automatic localization of anatomical regions, estimation of metacarpal-head cartilage thickness, and classification of synovial proliferation in metacarpophalangeal joints using ultrasound images. ML shows promise as a valuable tool for analyzing ultrasound images in diagnosing and assessing rheumatoid arthritis.	Ultrasonography	CNN	Hemalatha *et al*.^[24]^ (Apr. 2019)
		CNN	Fiorentino *et al*.^[25]^ (Feb. 2022)
		DL	Wu *et al*.^[26]^ (Feb. 2022)
Machine learning techniques applied to genetic data have identified immune-related biomarkers for rheumatoid arthritis, including diagnostic	Genetic data	SVM-RFE	Chen *et al*.^[27]^ (Nov. 2021)
signatures, immune cell infiltration characteristics, and specific markers		SVM-RFE, random forest	Yu *et al*.^[28]^ (Oct. 2021)
such as PSMB9 and CXCL13. Robust machine learning models have also			
been developed to predict RA using optimized polygenic risk scores. These findings advance our understanding of RA and demonstrate the		Linear regression, elastic Net, random forest	Rychkov *et al*.^[16]^ (Jun. 2021)
potential of machine learning in biomarker discovery.		Lasso, SVM-RFE	Li *et al*.^[29]^ (Dec. 2022)
		Bayesian	Lim *et al*.^[30]^ (Feb. 2023)
The studies demonstrate that ML methods, such as ABC analysis, random forest, logistic regression, and neural network, effectively differentiate	EHRs	Computed ABC analysis, random forest	Lötsch *et al*.^[31]^ (Jan. 2020)
between RA patients and non-RA individuals. These methods rely on utilizing EHR data, which includes clinical laboratory test results, demographic data, and clinical reports, *etc*. These studies highlight		SVM	Maarseveen *et al*.^[32]^ (Jun. 2021)
the potential of employing machine learning techniques and EHR to enhance the early diagnosis of RA.		SVM, logistic regression, and AdaBoost	Olatunji, *et al*.^[33]^ (Sep. 2022)
		ANN	Bai *et al*.^[14]^ (Jun. 2022)
		CSNN	Geng *et al*.^[34]^ (Aug. 2022)

CNN, convolutional neural network; SVM, support vector machine; DL, deep learning; DNN, deep neural networks; SVM-RFE, support vector machine-recursive feature elimination; AdaBoost, adaptive boosting; ANN, artificial neural network; CSNN, cost-sensitive neural network; EHRs, electronic health records; PSMB9, proteasome 20S subunit beta type-9; CXCL13, CXC chemokine ligand 13; EHR, electronic health record; ML, machine learning.

Furthermore, AI-based tools have been developed to analyze blood test results and clinical data from EHRs to assist in distinguishing RA from other inflammatory conditions. Many AI tools can help identify biomarkers that are indicative of RA and provide more accurate diagnoses. Mc Ardle *et al*.^[15]^ developed a random forest model that evaluated serum protein biomarkers and demonstrated good performance in discriminating between patients with RA and those with psoriatic arthritis. The model achieved an area under the curve (AUC) of 0.79 in the initial phase and 0.85 in the subsequent validation phase. The researchers created a robust matching learning feature selection pipeline, which identified new biomarkers for RA in over 2000 blood samples from RA patients.^[16]^

Overall, by utilizing AI in the diagnostic process, clinicians may be able to identify RA earlier, which can assist specialized physicians in developing tailored treatment strategies to prevent disease progression. This approach enables timely administration of antirheumatic drugs to achieve remission in rheumatoid arthritis patients. However, further research is needed to validate the accuracy and reliability of AI in diagnosing RA and to evaluate its feasibility and cost-effectiveness in clinical settings.

## AI in Early Intervention of RA

Early intervention in RA is important to slow down or even prevent the progression of joint damage and other complications associated with the disease, which can potentially bring benefits to both patients and society. Delayed treatment can result in irreversible joint damage, considerably impacting the patient’s ability to perform daily activities and causing long-term disability. Moreover, no treatment that has been demonstrated to be effective in preventing the development of RA, so a pressing requirement exists for the development of new biomarkers to initiate early intervention. Early treatment of non-steroidal anti-inflammatory drugs (NSAIDs) and disease-modifying anti-rheumatic drugs (DMARDs), which can slow or stop the progression of the disease and reduce joint damage. Frazzei *et al*.^[35]^ conducted a comprehensive systematic literature review and identified 1821 articles. The findings indicated that no treatment could prevent the onset of RA, but administering rituximab and abatacept at an early stage resulted in a delay in the onset of fully developed RA. Another research conducted by van der Linden *et al*.^[36]^ have demonstrated that starting treatment within the first 12 weeks of the onset of symptoms can lead to better outcomes, such as enhanced physical functioning and quality of life, as well as reduced rates of disability and joint damage. Therefore, treating RA early through appropriate interventions is crucial for improving long outcomes of the patient.

However, the early intervention of RA encounters various challenges, including the complexity of selecting appropriate medications, and the variability in medication response among individuals. In recent years, AI has shown the potential to improve early intervention of RA, as summarized in Table 2, which outlines various ML techniques applied in this context. As mentioned earlier, RA is an autoimmune disease that can affect the whole body, and usually affects joint first. However, the course of the disease can vary among individuals, and non-joint symptoms can be present at the early stages.^[37]^ Many ML methods have been investigated to detect disease progression.^[3]^ In a study conducted by Norgeot *et al*.,^[38]^ a longitudinal deep learning model was used to predict disease activity in RA using EHR data. Disease activity was classified using the clinical disease activity index (CDAI). The study demonstrated a strong predictive performance, with an area under the curve (AUC) of 0.91 in the test cohort. The most influential predictors for disease activity were found to be CDAI, cortisone injections, and C-reactive protein (CRP) levels.

**Table 2:** Summary of the studies of artificial intelligence (AI) in early intervention of rheumatoid arthritis (RA), including authors, data types, methods, and main findings of the same category

Main findings	Data types	Methods	Author (Month/Year)
ML techniques have demonstrated their value in the field of early intervention in RA by analyzing EHRs, clinical trial data and claims data. These	EHRs, claims data, clinical cohort, RCTs	DL	Norgeot *et al*.^[38]^ (Mar. 2019)
studies have used ML models to predict clinical		NLP	Spencer *et al*.^[42]^ (Nov. 2021)
outcomes, estimate disease activity scores, predict treatment response, identify predictors of severe COVID-19 outcomes, and cluster comorbidities in RA patients. These findings			
highlight the potential of ML to improve early intervention strategies for RA by leveraging		XGBoost, SVM	Morid *et al*.^[43]^ (Jul. 2021)
multiple healthcare data sources.		Random forests	Johansson *et al*.^[44]^ (May. 2021)
		Logistic regression	Burns *et al*.^[45]^ (Nov. 2022)
		Hierarchical clustering, factor analysis, k-means clustering, and network analysis	Crowson *et al*.^[46]^ (Feb. 2023)
		Factor analysis	England *et al*.^[47]^ (Feb. 2023)
		Linear regression, lasso and ridge, SVM, random forest, and XGBoost	Koo *et al*.^[48]^ (Jul. 2021)
		Logistic regression, k-nearest neighbors, naïve Bayes classifier and random forests	Vodencarevic *et al*.^[49]^ (Feb. 2021)
ML methods applied to genetic data in RA have demonstrated the potential to predict drug response, uncover molecular mechanisms of	Genetic data	GPR	Guan *et al*.^[50]^ (Dec. 2019)
therapy, identify genetic markers associated		Text mining	Wang *et al*.^[51]^ (Jul. 2021)
with clinical treatment response outcomes, to specific and treatments. accurately predict		Random forest, SVM	Kim *et al*.^[52]^ (Oct. 2021)
		Random forest	Tao *et al*.^[53]^ (Oct. 2021)
		Multivariate logistic regression, elastic net, random forest, and SVM	Kim *et al*.^[54]^ (Oct. 2022)
		Random forest	Lim *et al*.^[55]^ (Oct. 2022)
		Random forest	Lim *et al*.^[56]^ (Jan. 2022)
		Random forest	Myasoedova *et al*.^[40]^ (Jun. 2022)

DL, deep learning; NLP, natural language processing; SVM, support vector machine; GPR, Gaussian process regression; EHRs, electronic health records; RCTs, randomized controlled trials; XGBoost, eXtreme gradient boosting; COVID-19, coronavirus disease 2019; ML, machine learning.

Additionally, ML have been utilized to predict treatment responses to DMARDs, which classified into conventional synthetic disease-modifying antirheumatic drugs (csDMARDs), biologic disease-modifying antirheumatic drugs (bDMARDs) and targeted synthetic DMARDs (tsDMARDs).^[39]^ The research conducted by Myasoedova *et al*.^[40]^ is among the initial attempts to use machine learning techniques that combine clinical and genomic information to make personalized predictions about the response to methotrexate in individuals diagnosed with early RA. Along with baseline Disease Activity Score in 28 joints (DAS28) scores, intergenic single nucleotide polymorphisms (SNPs) such as rs12446816, rs13385025, rs113798271, and *ATIC* (rs2372536) demonstrated high importance (above 60.0) in predicting methotrexate response in RA patients. Yoosuf *et al*.^[41]^ used multi-omics analyses and ML models including linear model and kernel-based models to identify new biomarkers for the prediction response to anti-TNF treatment, which found pathways influenced by treatment. This information can provide valuable insights to physicians during evaluation and improve the decision-making process.

## AI in Disease Management of RA

Aside from early diagnosis and early intervention, proper management of RA is crucial to avoid long-term adverse outcomes, such as functional disability and increased mortality risk.^[57]^ In 2010, the European League Against Rheumatism (EULAR) published updated guidelines for the management of RA with DMARDs. These guidelines cover various aspects of RA management, including the use of conventional DMARDs, biologic DMARDs, and targeted synthetic DMARDs, as well as the timing and sequencing of treatments, monitoring of disease activity, and management of comorbidity.^[39]^ Effective and personalized management of RA is essential to enhance patients’ outcomes and quality of life.

However, poor treatment adherence, low compliance with follow-up appointments, and a lack of chronic disease management systems are common challenges in the effective management of chronic conditions such as RA.^[58]^ This can lead to sub-optimal outcomes, such as increased disease activity, joint damage, and functional disability, as well as reduced quality of life. Various factors that pose challenges to the effective management of RA, such as forgetfulness and misconception about the disease treatment at a patient level, and lack of patient education and support at a healthcare system level. The absence of a chronic disease management system poses a significant challenge in the effective management of RA. First, education is a critical component of early intervention, as it can help patients better understand their condition and the importance of adhering to treatment. In addition, regular monitoring and follow-up are important in early intervention, as they can help to ensure that patients are responding to treatment and that any changes in their condition are identified and addressed promptly.

Overcoming these barriers is crucial for effective RA management. In recent years, AI has shown a potential to improve the disease management of RA through the following avenues (Table 3). One application of AI in RA disease management is monitoring the disease, including scheduling follow-ups, notification for medications, *etc*. In the past decades, the field of mobile health (mHealth) has made significant advancements, resulting in a multitude of a smartphone (apps) designed for individuals with RA.^[59]^ Gossec *et al*.^[60]^ utilized data from wearable activity trackers that used machine-generated models for predicting flare sensitivity, and a connected monitoring interface on a smartphone developed by Pers *et al*.^[61]^ significantly lowered the number of physical visits. ML techniques have the ability to transform RA-related research and enhance disease management. However, these models are not currently prepared to fully contribute to daily practice, many issues like technique and ethical concerns with these methods need to be addressed before their implementation.^[62]^

**Table 3:** Summary of the studies of artificial intelligence (AI) in disease management of rheumatoid arthritis (RA), including authors, data types, methods, and main findings

Main findings	Data types	Methods	Author (Month/Year)
ML is being used in the field of RA to support dis-	Observational cohort	Bayes, random forests	Gossec *et al*.^[60]^ (Oct. 2019)
ease management. Applications include detecting flares based on physical activity data, predicting flares using ultrasound and blood test data,	Ultrasound images, blood test	Logistic regression, random forest, and XGBoost	Matsuo *et al*.^[63]^ (May. 2022)
extracting results from clinical notes using natural	EHRs	NLP	Humbert-Droz *et al*.^[64]^ (Mar. 2023)
language processing, and developing AI-based			
flare prediction systems. These approaches have the potential to improve disease monitoring in RA.	Clinical cohort	AI-powered RA clinical decision support tool	Labinsky *et al*.^[65]^ (Jan. 2023)

NLP, natural language processing; EHRs, electronic health records; XGBoost, eXtreme gradient boosting; ML, machine learning.

## Discussion

**Figure 1:**
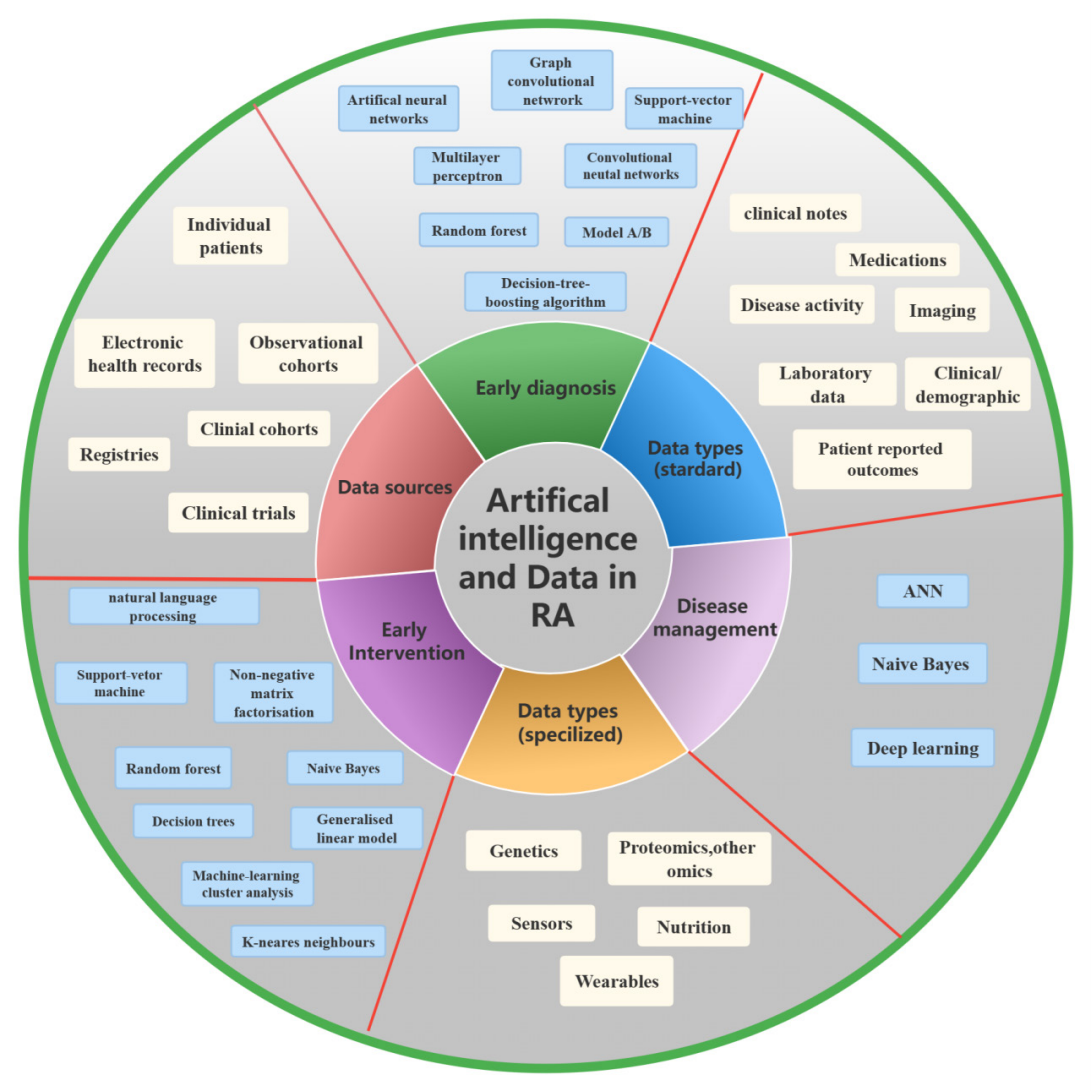
Artificial intelligence (AI) techniques, data types and data sources in early intervention, diagnosis and disease management in rheumatoid arthritis (RA); ANN, artificial neural network.

The objective of this study is to provide a concise overview of the role of AI in RA, specifically in the areas of early diagnosis, early intervention and disease management. Based on our overview, AI is a highly efficient and effective method for analyzing complex data in RA. We summarized various literature on key analyses, advanced methods, relevant strengths in these fields. As shown in Figure 1, ML has been widely used in the field of RA to improve diagnosis and treatment by using diverse types of data. EHRs, genetic data, and imaging data has been used to analyze and classify patients with RA, enabling more accurate and efficient diagnosis. ML techniques have also been used to predict treatment response using genetic data, enabling personalized and targeted interventions. By harnessing the power of ML, healthcare professionals can gain value insights from large datasets, identify patterns and make informed decisions about patients care. The integration of ML in RA has the potential to significantly improve patient outcomes and the overall management of the disease.

Despite the potential benefits of applying ML to RA, there are several barriers for wide-spread implementation. A major concern is the accuracy of the health data for ML analysis. Ensuring data quality is crucial for accurate results,^[62]^ but the available data from different sources maybe insufficient or inconsistent. In addition, standard class labels used for classification can be inconsistent between experts and can change over time as the understanding of RA evolves.^[66]^ Another significant obstacle is data security and privacy, particularly for sensitive information contained in EHRs. Decentralized data storage approaches, such as federated learning, have been proposed to minimize the impact of data breaches or hacking incident.^[67]^ However, there remains a risk of privacy attacks on the AI system. AI approaches have been explored in RA research, but few methods were practical in real medical settings. Achieving accurate diagnosis, early intervention and effective disease management in RA presents ongoing challenges.

Future studies of AI in RA should address above challenges. The establishment of a large-scale RA patient cohort with long-term follow-up may allow the development and validation of predictive models for the risk of occurrence and prognosis of major commodities, such as interstitial lung disease, cardiovascular disease, osteoporosis, and fragility fractures. The results of these model can inform the development of early warning strategies, and appropriate interventions, supporting the development of a comprehensive chronic disease prevention and management system for RA. In addition, due to the explosive growth of clinical data and the rapid development of AI, the development of an intelligent fusion analysis platform and the decision support system holds great potential for enhancing the precision and effectiveness of clinical diagnosis and treatment. However, further research is needed to fully develop and evaluate such a system.

## Conclusion

Overall, current AI has the potential to detect RA earlier, facilitate early intervention, and better disease management, but further research is needed to validate its accuracy and address ethical concerns.
